# Uptake of prevention of mother-to-child transmission cascade services in Burkina Faso between 2013 and 2020: are we on the right track?

**DOI:** 10.1186/s12905-023-02227-0

**Published:** 2023-03-24

**Authors:** Lucresse Corine Fassinou, Joël Ouoba, Calypse Ngwasiri, Issa Romba, Wedminere Noëlie Zoungrana-Yameogo, Fidèle Bakiono, Isidore Tiandiogo Traoré, Hervé Hien, Nicolas Nagot, Fati Kirakoya-Samadoulougou

**Affiliations:** 1 Institut Supérieur des Sciences de la Santé, Université Nazi Boni, Bobo-Dioulasso, Burkina Faso; 2 Centre for Research in Epidemiology, Biostatistics and Clinical Research of the School of Public Health, Université Libre de Bruxelles, Brussels, Belgique; 3 Secrétariat Permanent du Conseil National de lutte contre le Sida et les Infections Sexuellement Transmissibles, Ministère de la Santé, Burkina Faso; 4 Service d’information et d’épidémiologie, Centre Hospitalier Universitaire de Tengandogo, Ouagadougou, Burkina Faso; 5 Centre Muraz, Institut National de Santé Publique, Bobo-Dioulasso, Burkina Faso; 6 Pathogenesis and Control of Chronic and Emerging Infections, University of Montpellier, INSERM, University Antilles, Etablissement Français du Sang, Montpellier, France

**Keywords:** HIV, PMTCT indicators, Regions, Burkina Faso

## Abstract

**Background:**

The use of services to prevent mother-to-child transmission (PMTCT) of the human immunodeficiency virus (HIV) remains a serious challenge in sub-Saharan Africa. In the last decade, Burkina Faso has implemented numerous policies to increase the use of PMTCT services by pregnant women and their partners, as well as children. This study assesses trends in the uptake of PMTCT services in Burkina Faso from 2013 to 2020 in order to study the progress and gaps in achieving the national and international targets set for 2020.

**Methods:**

A repeated cross-sectional analysis was performed using data extracted from district health information software version 2. Percentages were computed for each PMTCT indicator and comparisons between the years were made using a chi-square test for trends with a significance threshold of 5%. Regions were not compared with each other.

**Results:**

The proportion of pregnant women who were tested and received their results significantly increased from 47.9% in 2013 to 84.6% in 2020 (*p* value < 0.001). Of the 13 regions in the country, only 1 region met the 95% national targets whereas, 6 regions met the 90% international targets for this indicator. The proportions of HIV-positive women receiving antiretroviral therapy (ART) increased from 90.8% in 2013 to 100% in 2020. In the same period, the proportion of exposed infants who received antiretroviral prophylaxis increased from 64.3% in 2013 to 86.8% in 2020. Only 3 regions reached the national and international targets for this indicator. A positive trend was also observed for the indicator related to screening at 2 months or later of exposed infants using Polymerase Chain Reaction (PCR) technic; with the rate rising from 7.4% in 2013 to 75.7% in 2020. However, for this indicator, the national and international targets were not achieved considering the national and regional settings. Concerning the women’s partners, the proportion of those who tested for HIV increased from 0.9% in 2013 to 4.5% in 2020, with only 1 region that fully met the national target of 10% in 2020. The prevalence of HIV in this particular group was 0.5% in 2020.

**Conclusions:**

PMTCT indicators show an increase from 2013 to 2020 but with a strong disparity between regions. National and international targets have not been achieved for any indicator; except for those related to women receiving ART. Strengthening strategies to effectively engage women and their partners on the use of PMTCT cascade services could help reduce mother-to-child transmission in Burkina Faso.

## Background

Globally, the incidence of paediatric Human Immunodeficiency Virus (HIV) infection has continued to decline, from around 320,000 in 2010 to about160,000 in 2020 [1]. More than 90% of these infections occur via mother-to-child transmission (MTCT), and in the absence of antiretroviral therapy (ART), roughly 50% of the infected children will die before their second birthday [1, 2]. Interventions during pregnancy, labour, delivery, and breastfeeding, collectively known as the prevention of mother-to-child transmission (PMTCT) cascade are effective in preventing the vertical transmission of HIV [3, 4]. The PMTCT cascade includes services aimed at virtually eliminating MTCT of HIV (Antenatal Care (ANC), HIV testing, disease staging, and ART), reducing child mortality (ARV prophylaxis, exclusive breastfeeding for the first 6 months, and Cotrimoxazole prophylaxis to prevent opportunistic infections), and improving maternal health [5].

Worldwide, around 1.4 million paediatric HIV infections were prevented from 2010 to 2018 because of PMTCT programs [6]. Also, because of PMTCT services, the proportion of children who acquired HIV infection from their mothers in some parts of Sub-Saharan Africa (SSA), plummeted from 18% in 2010 to 10% in 2017 [6]. In 2020, the worldwide expansion of access to PMTCT services resulted to 85% of pregnant women living with HIV who were receiving Antiretroviral Therapy (ART), preventing up to 220,000 new infants infections [4, 7]. Despite the global scale-up of PMTCT services, the pandemic continues to challenge maternal and paediatric services especially in SSA, where transmission through breastfeeding is responsible for more than half of all incident paediatric HIV infections and where many pregnant women miss some or all of the essential PMTCT services [8–10].

The prevention of HIV through PMTCT requires a multi-pronged approach which includes the HIV testing and treatment cascade for pregnant women and their infants [11]. The PMTCT cascade encompasses a series of key stepwise activities that constitute a critical pathway to successful PMTCT that begins with all pregnant women and ends with the detection of final HIV status in HIV-exposed infants [12]. It involves 18 months of care from the initial antenatal visit and HIV testing through ARV treatment, intra-partum care, infant testing, infant feeding education, and infant/mother treatment [13]. To further the global goal of the United Nations program on HIV/AIDS (UNAIDS) to eliminate MTCT by 2030, the World Health Organization (WHO) recommended the adoption of the option B + and suggested that ART should be initiated in all pregnant and breastfeeding women living with HIV, regardless of the clinical stage and CD4 cell count, and continued throughout their lives [14].

**Fig. 1:**
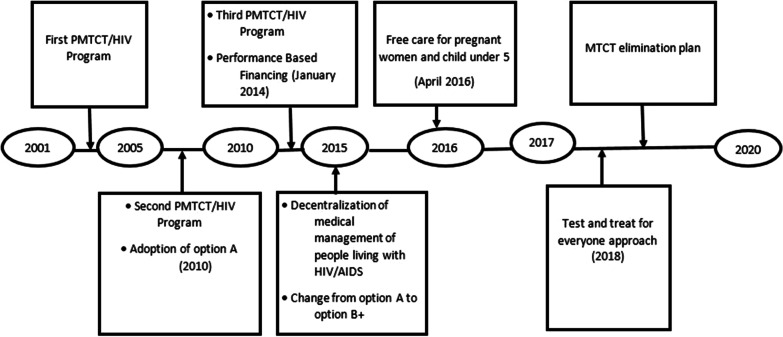
Timeline of different programs in connection with PMTCT adopted in Burkina Faso

Like many other countries in SSA, Burkina Faso has implemented several programs to boost PMTCT services and improve maternal and child health (Fig. 1) [15]. The first PMTCT/HIV program was implemented between 2001 and 2005, and covered 15% of all health facilities. The second program was executed from 2006 to 2010 in 92% of the health facilities, and the third was carried out from 2011 to 2015 with effective implementation in 98.2% of the health facilities [16, 17]. The latter program integrated maternal health services with HIV testing and free ARV treatment [16]. Additionally, the option B + approach was effectively implemented in 2015, as was the decentralisation of the medical management of people living with HIV and the delegation of prescriptions to paramedics so that national and international targets could be achieved [18, 19].

The objectives of national targets aiming at the elimination of MTCT by the end of 2020 included testing of 95% of pregnant women for HIV during ANC, ARV treatment of 95% of HIV-positive pregnant women to reduce the risk of transmission, HIV testing in 10% of male partners, complete HIV testing in 60% of women of childbearing age using family planning services, HIV testing in the first six weeks of 95% of children born to HIV-positive mothers, and ARV prophylaxis for 95% of children born to HIV-positive mothers [17]. Internationally, the UNAIDS and its partners set the ‘90–90–90 targets’ which aimed at testing 90% of all people living with HIV, treating 90% of those infected, and achieving viral suppression in 90% of those on treatment by 2020 [20, 21].

As Burkina Faso is in the process of eliminating MTCT of HIV, an estimation of the coverage of PMTCT services is vital to monitoring the progress related to both national and international targets, and to secure donor funding for PMTCT programs [22, 23]. Thus, the main purpose of this study was to assess trends in the uptake of PMTCT services from 2013 to 2020 in order to identify the roadblocks to achieving the national and international targets set for 2020. The findings could help improve interventions to reach the 2030 objective of elimination of HIV in Burkina Faso.

## Methods

### Data source

This was a retrospective, repeated cross-sectional analysis of aggregated health facility data from the National Data Warehouse of the Burkina Faso Ministry of Health (ENDOS-BF), a district health information software version 2 (DHIS2) web platform which has been operational since 2013. The National Ethics Committee “Comité d’Ethique pour la Recherche en Santé (CERS)” of Burkina Faso (n°2021-06-157) approved the study protocol. Given that the analysis was performed at the population level without reference to information that would allow the identification of participants, the requirement for individual informed consent was waived by the Ethics Committee. The study was conducted with the CERS authorization and all methods were carried out in accordance with its requirements.

In 2019, due to a prolonged strike by healthcare personnel including the retention of health data, some data were not available for use in this study. The indicators on the use of PMTCT services and their calculations are presented in Table 1.

**Table 1:** Description of the indicators used to assess the uptake of PMTCT services in Burkina Faso from 2013 to 2020

Indicators	Numerator	Denominator
*Pregnant women*		
Proportion of pregnant women who were tested for HIV and received their results	Number of pregnant women who were tested for HIV and received their results	Number of new ANC
Proportion of pregnant women tested positive for HIV during ANC	Number of pregnant women who tested positive for HIV during ANC	Number of pregnant women who tested for HIV and received their results
Overall proportion of pregnant women who were HIV positive during ANC	(Number of pregnant women who tested positive for HIV during ANC) + (number of pregnant women already known to be HIV positive)	Number of new ANC
Proportion of pregnant women who were HIV positive delivering in the health facility	Number of pregnant women who were HIV positive delivering in the health facility	Number of pregnant women who tested positive for HIV
Proportion of pregnant women who were HIV positive and benefitted from post-partum family planning	Number of pregnant women who were HIV positive and benefitted from post-partum family planning	Number of pregnant women who were HIV positive delivering in the health facility
Proportion of pregnant women who were HIV positive and receiving ART	Number of pregnant women who were HIV positive and receiving ART	Number of pregnant women who tested positive for HIV
*Partners of pregnant women*		
Proportion of partners of pregnant women who were tested for HIV	Number of partners of pregnant women who were tested for HIV	Number of new ANC
Proportion of partners of pregnant women who were tested HIV-positive	Number of partners of pregnant women who tested HIV-positive	Number of new ANC
Proportion of women of childbearing age using family planning services who completed the voluntary HIV test	Number of women of childbearing age using family planning services who completed the voluntary HIV test	Number of women of childbearing age using family planning services
*Infants born to HIV-positive mothers*		
Proportion of infants born to HIV-positive mothers receiving full ARV prophylaxis	Number of infants born to HIV-positive mothers receiving full ARV prophylaxis	Number of infants born alive to HIV-positive mothers
Proportion of infants born to HIV-positive mothers who received cotrimoxazole prophylaxis	Number of infants born to HIV-positive mothers who received cotrimoxazole prophylaxis	Number of infants born alive to HIV-positive mothers
Proportion of infants born to HIV-positive mothers who were screened at 2 months of life or later with Polymerase Chain Reaction (PCR) technic	Number of infants born to HIV-positive mothers who were screened at 2 months of life or later with PCR technic	Number of infants born alive to HIV-positive mothers
Proportion of infants who were tested HIV-positive via PCR technic	Number who tested HIV-positive via PCR technic	(Number of infants born to HIV-positive mothers who were screened at 2 months of life with PCR technic) + (Number of infants born to HIV-positive mothers who were screened after 2 months of life with PCR technic)
Proportion of infants born to HIV-positive mothers who were screened with rapid tests at 18 months of life	Number of infants born to HIV-positive mothers who were screened with rapid tests at 18 months of life	Number of infants born alive to HIV-positive mothers
Proportion of infants who were tested HIV-positive with a rapid diagnostic test at 18 months of life	Number of infants tested HIV-positive with a rapid diagnostic test at 18 months of life	Number of infants who received rapid diagnostic test at 18 months of life

For early infant diagnosis, the PCR test was performed at 2 months of age. When this test was not performed at 2 months, it was carried out at a later date, and whether or not the result was positive, a rapid serological test was planned at 18 months post-partum (end of the risk of postnatal transmission of HIV). The data obtained for this indicator were only related to screening and did not consider the receipt of the results. Data concerning PCR testing after 2 months were not available for the years 2013, 2014, and 2015.

### Setting

Burkina Faso is a landlocked Sahelian country with a surface area of 272,967 km^2^ (Institut Géographique du Burkina IGB, 2002) and is bordered by six countries in the West African sub-region (Mali, Niger, Benin, Togo, Ghana, and Côte d'Ivoire) [24]. It is divided into 13 administrative regions and has 45 provinces [25].

In 2020, Burkina Faso had 06 university teaching hospitals, 09 regional hospitals, 46 medical centres with surgical facilities, 71 medical centres, 2041 health and social promotion centres, 111 isolated dispensaries, 09 isolated maternities and 641 private health facilities (hospital and non-hospital) [26]. Based on the activity report on the AIDS response in Burkina Faso in 2019, a total of 100 [public (75), private (9), faith-based (6), and community (10)] sites provided healthcare support (ART medical management) to persons living with HIV throughout the 13 regions of the country [27]. In addition to the coverage of all health districts, 1820 out of 1853 health facilities provided PMTCT services (coverage rate of 98.2%) [24]. Despite this high coverage, the enrolment rate of pregnant women for PMTCT services was 86.1%, which was still below the national target of 90% [24].

### Statistical analysis

Each indicator was computed as a proportion, and comparison was made between the different years using a chi-squared test for trends. Regions were not compared with each other. The significance threshold was set to 5% and all analysis was done with STATA version 16 software.

## Results

**Fig. 2:**
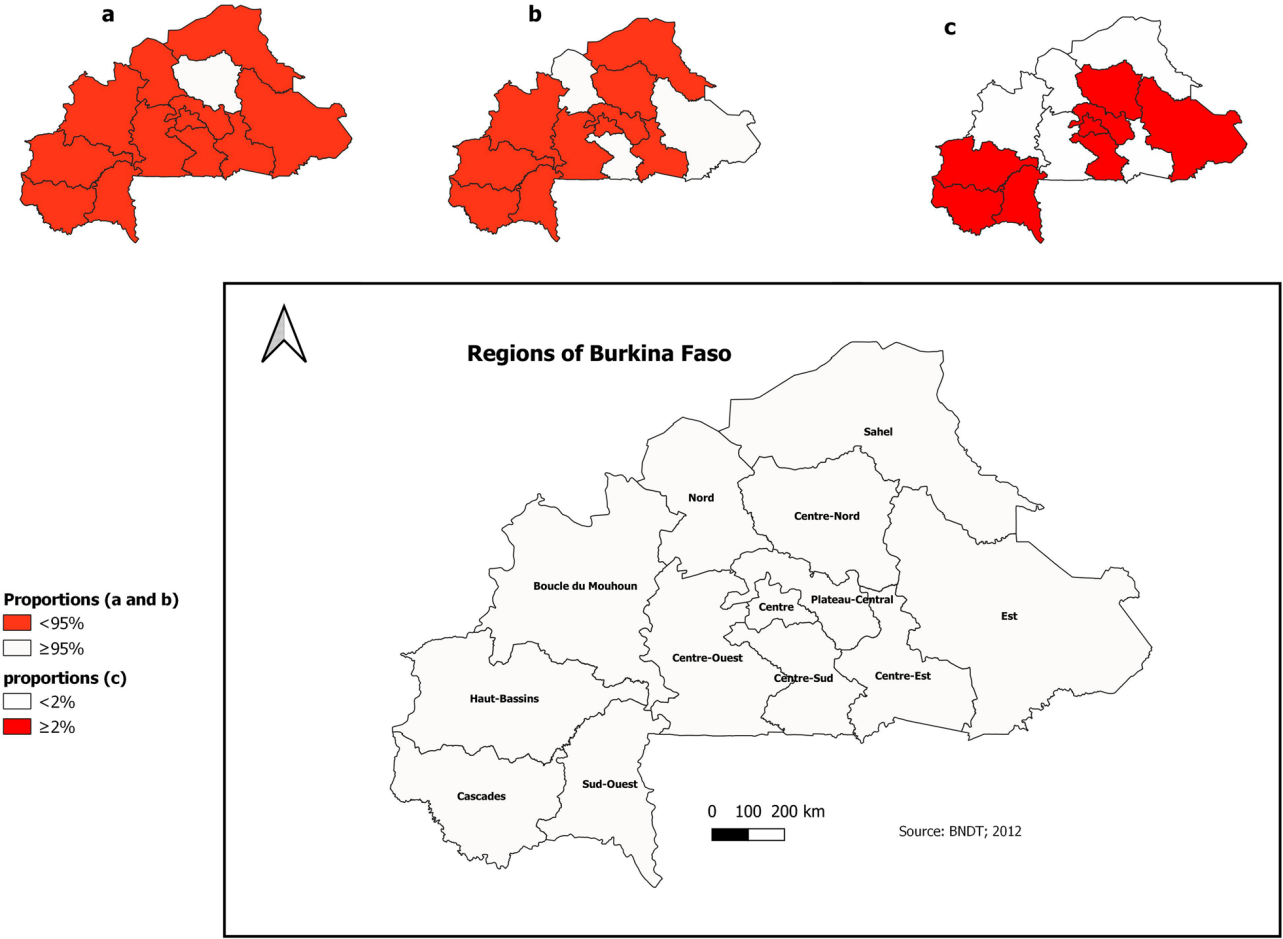
Proportions of the main indicators of PMTCT in Burkina Faso in 2020. The proportion of pregnant women who tested for HIV during ANC and who received their results (**a**); the proportion of infants on ARV prophylaxis (**b**); and the proportion of exposed infants tested HIV-positive with PCR technic (**c**) is indicated

The proportion of pregnant women who were tested and received their results significantly increased from 47.9% in 2013 to 92.2% in 2018, and then decreased to 84.6% in 2020 (*p* value  < 0.001, Table 2). Only one region (Centre-Nord) met the national target of 95% of women who were tested for HIV and received their results (Fig. 2a), and six regions (Boucle du Mouhoun, Centre, Centre-Nord, Centre-Ouest, Centre-Sud, Nord) met at least the international target of 90%.

**Table 2:** Trend of the PMTCT cascade indicators between 2013 and 2020 in Burkina Faso

Indicators	2013	2014	2015	2016	2017	2018	2019	2020	*p* value
Proportion of pregnant women who were tested for HIV and received their results (%)	47.9	83.4	85.3	84.9	84.0	92.2	88.1	84.6	< 0.001**
Proportion of pregnant women tested positive for HIV during antenatal care (ANC) (%)	1.7	0.9	0.8	0.7	0.7	0.6	0.6	0.5	< 0.001**
Overall proportion of pregnant women who were HIV positive during ANC (%)	8.1	3.6	2.0	1.5	1.4	1.3	1.5	1.1	< 0.001**
Proportion of pregnant women who were HIV positive delivering in the health facility (%)	39.3	53.5	51.8	45.4	51.3	49.4	NA	62.7	< 0.001**
Proportion of pregnant women who were HIV positive and benefitted from post-partum family planning (%)	NA	NA	NA	63.3	89.0	52.1	NA	NA	0.029*
Proportion of partners of pregnant women who were tested for HIV (%)	0.9	1.2	1.8	2.5	2.8	3.2	NA	4.5	< 0.001**
Proportion of partners of pregnant women who were tested HIV positive (%)	0.1	0.3	0.6	0.4	0.6	0.4	NA	0.5	< 0.001**
Proportion of women of childbearing age using family planning services who completed the voluntary HIV test (%)	10.6	6.5	10.8	13.4	11.7	11.6	NA	17.2	< 0.001**
Proportion of pregnant women who were HIV positive and receiving ART (%)	NA	NA	NA	90.8	100	96.3	100	100	< 0.01*
Proportion of infants born to HIV-positive mothers receiving full ARV prophylaxis (%)	64.3	70.4	73.1	94.4	93.4	93.4	86.8	86.8	< 0.001**
Proportion of infants born to HIV-positive mothers who received cotrimoxazole prophylaxis (%)	50.9	53.3	66.9	78.0	73.4	82.6	NA	74.4	< 0.001**
Proportion of infants born to HIV-positive mothers who were screened at 2 months of life or later with PCR technic (%)	7.4	39.3	48.5	55.0	59.4	57.9	63.6	75.7	< 0.001**
Proportion of infants who were tested HIV-positive via PCR technic (%)	22.8	10.0	7.9	8.1	5.3	3.2	NA	3.8	< 0.001**
Proportion of infants born to HIV-positive mothers who were screened with rapid tests at 18 months (%)	21.0	25.5	28.7	22.9	25.1	25.3	32.1	26.9	< 0.001**
Proportion of infants who were tested HIV-positive with a rapid diagnostic test at 18 months of life (%)	12.9	14.7	8.4	31.6	8.5	6.8	9.1	9.3	< 0.001**

*NA* not available

*Means significant *p* value (< 0.05); **means a very significant *p* value (< 0.001)

Amongst the pregnant women who were tested for HIV, about 2 women in 100 were tested HIV-positive in 2013, compared to 5 in 1000 in 2020 (*p* value  < 0.001). The annual incidence was under 2% in 2020, with the highest incidence occuring in the Centre region (1.0%) and lowest in the Est region (0.1%).

The proportion of HIV-positive pregnant women who received ART also increased from 90.8% in 2016 to 100% in 2020. Likewise, the proportion of infants born to HIV-positive mothers receiving full ARV prophylaxis increased from 64.3% in 2013 to 86.8% in 2020.

Screening at 2 months or later with PCR technic in infants significantly increased from 7.4% in 2013 to 75.7% in 2020 (*p *value <0.001). At the regional level, the rate of infants receiving full ARV prophylaxis was at least 95% in 3 regions (Centre-Sud, Est, and Nord) in 2020 (Fig. 2b).

Amongst infants screened with PCR technic, the proportion of those who were tested HIV-positive decreased from 22.8% in 2013 to 3.8% in 2020. The lowest prevalence in 2020 was observed in the Centre-Est and Sahel regions, where no child were tested HIV-positive using PCR technic. In addition to these two regions, less than 2% of the exposed infants in 3 other regions (Boucle du Mouhoun, Centre-Ouest, and Nord) were tested HIV-positive in 2020 using PCR technic (Fig. 2c). Furthermore, in 2020, a high rate of HIV-positive exposed infants was observed in the Est and Plateau-Central regions via PCR technic (7.5% and 9.8%, respectively) and via rapid diagnostic testing at 18 months (71.4% and 13.3%, respectively).

Regarding the partners of the pregnant women, the proportion of those who were tested for HIV increased from 0.9% in 2013 to 4.5% in 2020, with a prevalence of HIV of 0.5% in 2020. Centre-Nord was the only region to meet the national target (10%) of the proportion of partners tested for HIV in 2020 (15.9%).

## Discussion

This study aimed to assess trends in the uptake of PMTCT services in Burkina Faso from 2013 to 2020. Our findings show that the proportion of pregnant women tested for HIV during ANC and who received their results, and HIV positive pregnant women receiving ART, significantly increased over the years. Positive trends were also observed for exposed infants who received ARV prophylaxis; those who performed a PCR screening at 2 months or later; and the proportion of partners of the pregnant women who were tested positive for HIV. National targets for HIV testing in pregnant women were achieved in just 1 region whereas 6 regions met the required international targets for testing and treatment.

Our study suggests that Burkina Faso has made significant improvements in the uptake of PMTCT services from 2013 to 2020. Despite this progress, gaps remain in the uptake of PMTCT services amongst pregnant women and infants in relation to the national and international targets that were not achieved for most indicators, except for the proportion of pregnant women receiving ART in 2020.

Implementing a strategy to reduce vertical HIV transmission involves two steps: testing women infected with HIV and accessing coordinated care for women and their children [28]. In terms of screening, the proportion of pregnant women who were tested and received their results during ANC increased by more than 50% from 2013 to 2020, showing that pregnant women have strongly adhered to PMTCT screening services in Burkina Faso. These results are consistent with findings from a similar study in Nigeria, which showed an increase from 50.1% in 2013 to 70.5% in 2017 [29]. This positive trend could be explained by the introduction of free HIV testing and ART in 2010 and the integration of HIV testing into reproductive health care in 2013 [30] associated with adequate counselling [31] given during ANC and sensitisation by community-based health workers through home visits [32–34].

However, the rate slightly decreased in 2019 and 2020 respectively. The decrease in 2019 could be explained partly by the repeated strikes conducted by healthcare workers that year that lasted 8 months and were mainly motivated by wages increases, notably the application of the law on hospital services [35]. It could also be explained by the political instability the country has been facing and that worsened in 2019 with violent attacks that resulted in the closure or minimal operation of more than 200 health centres, limiting access to health services for around 1.2 million of persons [36]. A plethora of studies have revealed that political instability negatively impact maternal healthcare services utilization [37–39], and in the particular context of Burkina Faso, it has been shown that the worsening security situation led to a reduction in ANC visits by 1.8% [40]. Another reason for the decline in the proportion of pregnant women tested for HIV in 2020 was due to the disruptive effects of the coronavirus-19 (COVID-19) pandemic on the healthcare system which may have impacted ANC and healthcare facility attendance due to the fear of contracting the virus. Results from several studies conducted in Africa revealed a decline in the uptake of healthcare services by pregnant women and infants after the pandemic began [41–43].

To better understand the impact on the uptake of PMTCT services and to identify innovative approaches to improve both the provision and utilisation of healthcare in this epidemiological context, both qualitative and quantitative studies are needed so that adapted measures based on the results can be quickly applied, avoiding a breakdown of the efforts made thus far to eliminate the MTCT of HIV in Burkina Faso. In addition, strengthening current strategies is necessary to bring this indicator up to the national target and maintaining it at this level. In 2020, only one region met the national target of 95% of the proportion of pregnant women tested for HIV and who received their results by the year 2020 [17]. The global UNAIDS and WHO targets of 90% were not achieved in 2020 (current proportion of 84.6%) showing a low uptake of this service. Strong efforts are thus required in low-performing regions to improve the rate of this indicator to achieve both national and international targets.

Amongst the pregnant women who received ANC between 2013 and 2020, the prevalence of HIV decreased from 8.1% in 2013 to 1.1% in 2020, with a cumulative incidence ranging from 1.7% in 2013 to 0.5% in 2020. This decrease could be explained by the establishment of voluntary counselling and testing services for HIV among pregnant women in Burkina Faso [44]. In 2015, Burkina Faso adopted the option B+, which entails lifetime combined ART in pregnant women and ARV prophylaxis in new-borns [19].

The ART uptake of pregnant women significantly increased from 90.8% in 2016 to 100% in 2020 whilst ARV prophylaxis in exposed infants also increased from 64.3% in 2013 to 94.4% in 2016 but later decreased to 86.8% in 2020. The national target of 95% [17] was nearly achieved in 2016, and a decline was observed in 2019 and 2020 which was lower than the international targets of 90%. This trend could be explained by the reduction in the proportion of women who were tested and received their results, as well as the fact that the WHO option B+ focuses heavily on maternal ART, whereas infant pre-exposure prophylaxis (PrEP) was a lower priority. By testing more women, most of them would know their HIV status, and more exposed infants would also benefit from ARV prophylaxis.

The proportion of pregnant women who tested for HIV and received their results globally increased between 2013 and 2020 but a slight decrease was observed between 2015 and 2017. This decrease could be explained by factors such as: the change of strategy (the transition from option B to option B+ in 2015) which could have impacted the provision of services by the healthcare facilities and the demotivation of healthcare workers due to long delay in the payment of Performance-based financing (PBF) bonuses for the achievement of quantitative objectives [45]. Studies done in Burkina Faso showed great acceptability in the testing uptake by pregnant women but low rates in the reception of testing results [46].

Regarding regional disparities, only 3 regions (Centre-Sud, Est, and Nord) met the national target of 95% for this indicator, suggesting that the policy in place did not work well in the other regions and that targeted interventions should be taken to improve and reach national targets related to reducing MTCT. Concerning the exposed infants, a significant increase was globally observed from 2013 to 2020 but however decreased in 2019, may be due to the health crisis caused by the political instability the country has been facing; which could have acted successively on demand (services no longer being frequented by mothers and children because of the absence of health workers in certain treatment sites….), and the provision of services (access to basic services such as testing services, delivery of results, care of mothers born with HIV,..).

Our results also showed an increase in the proportion of exposed infants tested at 2 months or later with PCR technic, up to 75.7% in 2020. However, despite this increase, the result is still far from the international and national targets of 90% and 95%, respectively. PCR screening is an important factor for the early determination of children’s status and allows healthcare providers to offer optimal care in the management of HIV, assist in decision making regarding infant feeding, and avoid needless stress for mothers and families, helping them increase the efficacy and coverage of PMTCT interventions [47]. Similar studies conducted in SSA have also observed this trend of a low rate of achievement of PCR testing [48, 49]. This poses a real problem with the ARV start-up protocol in HIV-infected infants and may be the basis for the increase in mortality due to HIV [50]. At 18 months of age, we observed a relatively low rate of less than 50% among exposed infants from 2013 to 2020. Although 75.7% of the exposed infants underwent PCR testing at 2 months or later in 2020, only 26.9% did a rapid diagnostic test at 18 months, showing a low uptake of services for HIV-exposed infants, and indicating that there is a gap in the use of the PMTCT cascade, which constitutes a barrier to the success of the program. According to the WHO’s recommendation, after ‘the early infant diagnosis’ at 6 weeks, another HIV test should be carried out at 18 months to provide the final infant diagnosis with infant infections that are being transmitted during breastfeeding [51]. HIV-positive infants and children who start treatment late are more likely to experience treatment failure, thus, highlighting the need for early diagnosis and emphasizing the importance of the final testing at 18 months, which marks the end of exposure to postnatal transmission of HIV[51].

We also observed that most pregnant and postpartum women are not completely removed from the cascade despite most of the results showing a suboptimal uptake of services in the cascade. For example, in 2020, 84.6% of pregnant women were tested for HIV and received their results. Among those who tested HIV positive, 100% of them received ART, and 86.6% and 74.4% of their infants were administered full ARV and cotrimoxazole prophylaxis respectively. Almost 75.7% of these infants were screened at 2 months or later with PCR technic, but only 26.9% of them received final testing at 18 months. Our results are consistent with those of several other studies [52, 53] but show that efforts are still needed; first, to improve the uptake of PMTCT services and secondly, to concentrate more on the postpartum period to ensure a continuum of care.

Another factor that could affect the success of the PMTCT program is partner testing. According to an activity report on the AIDS response in Burkina Faso, partner screening is an indicator of the involvement of men in reproductive health, particularly regarding PMTCT [24]. However, our study revealed that only 4.5% of partners were screened in 2020, whereas the national target was 10%. Only one region met this target, indicating a strong disparity between regions as well as the low engagement of partners in PMTCT services, which could have negatively impacted the follow-up of the mother/child pair [54].

Our results should be interpreted with caution. Data included in the analyses were aggregated and as a result, information bias could have occurred while collecting or compiling data in each healthcare facility, as well as missing data among some relevant indicators. However, these aggregated data are sufficiently powerful to give reliable information. Since these data do not include individual or longitudinal information on women, partners, or mother-baby pairs through the cascade of PMTCT events, the rate of women and children retained in care (which is an important measure for the assessment of the PMTCT program) could not be determined. Although our study sort to assess the uptake of different services constituting the cascade of PMTCT, these variables were not available in our analysis. Therefore, further research involving the triangulation of data could include this aspect to get a deeper and more comprehensive information on the uptake of PMTCT services in Burkina Faso. It should also be noted that many indicators were not collected in 2019 which did not allow us to better appreciate the tendency for those indicators.

Regardless of these limitations, our results suggest that strong monitoring efforts must be made to maintain a high number of HIV-positive women undergoing ART and to improve the screening of infants born to HIV-positive mothers at 2 and 18 months to achieve the goal of eliminating the vertical transmission of HIV in Burkina Faso. The screening of partners and ARV prophylaxis of infants born to HIV-positive mothers are indicators that should also be improved.

## Conclusion

PMTCT indicators improved over time in Burkina Faso; however, strong disparities remain between regions. Despite these improvements in PMTCT services, only 1 indicator related to HIV-positive pregnant women receiving ART met the national and international targets set for 2020. In the era of the next UNAIDS 95-95-95 target, and the context of low coverage of PMTCT indicators in some regions, strategies to address these disparities are needed to achieve the targets set for 2030 in Burkina Faso.

## Data Availability

The data used in this study is not publicly available. It can be available from the Directorate General of Studies and Sectorial Statistics of the Ministry of Health of Burkina Faso at the e-mail: contact@sante.gov.bf.

## Abbreviations

AIDS: Acquired immunodeficiency syndrome
ANC: Antenatal care
ART: Antiretroviral therapy
ARV: Anti-retroviral
CD4: Cluster of differentiation 4
CERS: National Ethics Committee of Burkina Faso
DHIS2: District health information software version 2
ENDOS-BF: National Data Warehouse of the Burkina Faso Ministry of Health
MTCT: Mother-to-child transmission
PCR: Polymerase chain reaction
PMTCT: Prevention of mother-to-child transmission
PrEP: Pre-exposure prophylaxis
SSA: Sub-Saharan Africa
UNAIDS: United Nations Programme on HIV/AIDS
VIH: Human immunodeficiency virus
WHO: World Health Organization
